# The ∼16 kDa C-Terminal Sequence of Clathrin Assembly Protein AP180 Is Essential for Efficient Clathrin Binding

**DOI:** 10.1371/journal.pone.0110557

**Published:** 2014-10-20

**Authors:** Ling-Shan Chan, Lia Moshkanbaryans, Jing Xue, Mark E. Graham

**Affiliations:** Children’s Medical Research Institute, The University of Sydney, Westmead, New South Wales, Australia; University of Edinburgh, United Kingdom

## Abstract

Brain-specific AP180 is present in clathrin coats at equal concentration to the adapter complex, AP2, and assembles clathrin faster than any other protein *in vitro*. Both AP180 and its ubiquitously expressed homolog clathrin assembly lymphoid myeloid leukemia protein (CALM) control vesicle size and shape in clathrin mediated endocytosis. The clathrin assembly role of AP180 is mediated by a long disordered C-terminal assembly domain. Within this assembly domain, a central acidic clathrin and adapter binding (CLAP) sub-domain contains all of the known short binding motifs for clathrin and AP2. The role of the remaining ∼16 kDa C-terminal sequence has not been clear. We show that this sequence has a separate function in ensuring efficient binding of clathrin, based on *in* *vitro* binding and *ex* *vivo* transferrin uptake assays. Sequence alignment suggests the C-terminal sub-domain is conserved in CALM.

## Introduction

Clathrin mediated endocytosis (CME) occurs in all eukaryotic cells [1]. Multiple endocytic modes occur at presynaptic nerve terminals, but CME is the most well understood [2]–[5]. Synaptic vesicles are generated by CME using brain specific proteins that are homologous to the non-neuronal CME protein machinery [4]. CME involves the formation of lattice-like “pits”, which mature into “baskets” and “cages” made from clathrin triskelia during vesicle budding [6]. Clathrin cannot directly bind lipid membrane. Thus, clathrin is recruited to the membrane by adaptor proteins or protein complexes. The adapter protein complex 2 (AP2) is frequently referred to as the main clathrin adapter at the plasma membrane [7]. The AP2 complex is present in approximately equal concentration with each clathrin triskelion in clathrin coated vesicles. AP2, has a well-defined structure [8]–[10] and functions as an endocytic protein-protein interaction hub [11]. However, another adapter, assembly protein 180 (AP180) [12], is also present in equal concentration to clathrin triskelia in coated vesicles [13]. AP180 has a ubiquitously expressed homolog, clathrin assembly lymphoid myeloid leukemia protein (CALM), which is equally abundant as AP2 in clathrin coated vesicles from HeLa cells [14]. AP2 was known to assemble clathrin *in* *vitro* [15]. AP180 was shown to have four times the clathrin assembly activity of AP2 *in* *vitro* [16] and AP180 cooperatively assembled clathrin with AP2 faster *in* *vitro* than either protein alone [17]. However, the AP2 *in* *vitro* clathrin assembly activities are in doubt because of recent work, which has shown that AP2 is auto-inhibited from recruiting or assembling clathrin until it undergoes a membrane-mediated conformational change which regulates the availability of a clathrin binding motif (CBM) [18]. Despite that AP180 and CALM are highly abundant components in their respective CME roles, there is no similarly detailed mechanism of AP180 or CALM binding to clathrin, as there is with AP2.

When *Drosophila* (lap) or *C. elegans* (unc-11) homologs of AP180 and CALM were knocked out, synapses failed to generate a sufficient number of synaptic vesicles, the probability of neurotransmitter release was reduced and the remaining synaptic vesicles were abnormally large and deformed [19], [20]. AP180 assembles clathrin cages *in* *vitro* which have a narrow size distribution [21], [22]. Depletion of CALM in HeLa cells also led to irregular shaped budding vesicles [23]. However, this phenotype is not unique to AP180 homologs. Knockout of a component of the vesicle fusion machinery, synaptobrevin 2/vesicle associated membrane protein 2 (VAMP2) [24], as well as other synaptic vesicle proteins, also leads to large and deformed synaptic vesicles. In 2011, CALM was shown to sort VAMP2 into vesicles via binding to the AP180 N-terminal homology (ANTH) domain [25], [26]. This raised the question of whether the irregular vesicle phenotype is a consequence of an assembly defect or improper VAMP sorting, although no mechanism for the latter has been proposed. This question was recently addressed by Sahlender *et al.* [27] by using CALM mutants that cannot bind VAMP. The wild type CALM and VAMP defective mutants were able to rescue the morphological changes to clathrin coated pits. Therefore, VAMP binding is likely to have no influence on the role of CALM and AP180 in controlling clathrin coated vesicle size and shape. Thus, AP180 and CALM have evolutionarily conserved functions in both VAMP sorting and controlling vesicle size and shape and current evidence indicates these functions are independent of each other.

AP180 is proposed to act as the primary driver of clathrin cage assembly [28]. This is supported by its primary structure which contains more CBMs than any other protein [29]. The Lafer group identified 12 putative CBMs in AP180 (most conform to a consensus of D(L/I)(L/F)), demonstrated the functional importance of these CBMs for assembly *in* *vitro* and showed that introduced CBM peptides block endocytosis [29], [30]. These 12 CBMs, were hypothesised to be a variation or degenerate version of the conventional CBM, L(L/I)(D/E/N)(L/F)(D/E) [31], or the revised consensus CBM, pLΦpΦp [32] (where p = polar and Φ = hydrophobic). Clathrin assembly by AP180 fragments *in* *vitro* is dependent on the number of CBMs, implying that these motifs confer multi-valent clathrin binding and assembly properties [29]. A structural study of an AP180 peptide (623–680) containing two CBMs found that each CBM was locally structured and had a similar weak affinity for the clathrin heavy chain N-terminal domain (K_d_ ∼250 µM) [30]. This data on AP180 CBMs has given rise to the “fishing line of baited hooks” model for AP180, where multiple CBMs with weak binding affinity can efficiently recruit and assemble clathrin [30], [33], [34]. Multiple AP180 CBMs potentially interact with multiple clathrin heavy chain N-terminal domains to “tighten” the clathrin cage and produce the small synaptic vesicles found in neurons [29]. This model explains the relationship between AP180 CBMs and assembly function, but evidence is lacking for a role in clathrin recruitment and the assumption that the CBMs are equally available and wholly responsible for binding does not fit the available evidence (see below).

CALM has a single DLL CBM and one AP2 binding DIF sequence [23], which would potentially limit the ability of CALM to tighten cages via CBMs. However, previous work with truncated CALM sequences revealed there are unidentified CBMs in CALM in the C-terminal ∼150 amino acid (aa) residues and they are required for transferrin (Tfn) uptake [35], [36]. Recently, a conserved leucine-rich sequence in this C-terminal sub-domain was found to be a functional nuclear export sequence for CALM [37], [38], demonstrating there are more functions to be discovered in this tail region. The ∼16 kDa C-terminal sub-domain of AP180 currently has no clear function. This sequence was previously found to bind clathrin cages and very weakly assemble clathrin, but has no D(L/I)(L/F) CBM [29], [39]. Furthermore, the isolated CLAP domain bound clathrin triskelia weakly and could not assemble clathrin [39], whereas the CLAP combined with the ∼16 kDa C-terminal sub-domain assembles clathrin as well as full length AP180 [39]. Thus, the mechanism by which these sub-domains combine to efficiently bind and assemble clathrin has not been adequately explained.

We have examined N- and C-terminal truncated AP180 sequences using pull-down experiments and a Tfn uptake assay to further investigate the role of AP180 CBMs and the C-terminal sub-domain in clathrin binding and CME. We demonstrated that clathrin binding does not correlate with the number of CBMs in each AP180 fragment we examined. The binding of clathrin to particular fragments suggested that additional binding or inhibitory elements mediate the clathrin-AP180 interaction. Progressive C-terminal truncation of the ∼16 kDa sub-domain abolished the ability of AP180 to bind clathrin, demonstrating its crucial role in clathrin binding.

## Results

### Binding of clathrin to AP180 sequences does not correlate with known CBMs

**Figure 1:**
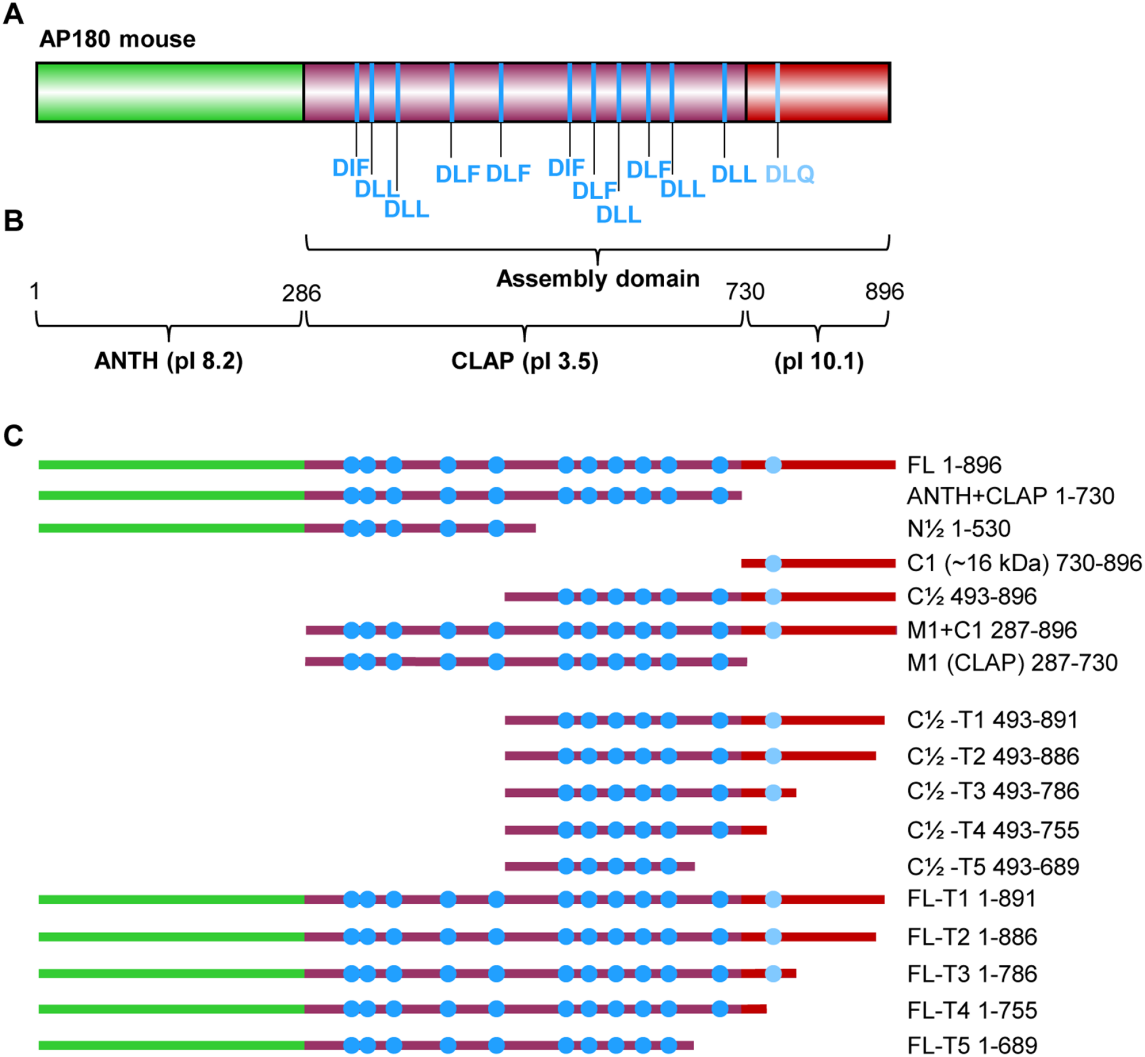
AP180 domain structure and recombinant proteins used in this study. (A) Domain structure of AP180 (Mouse, short, isoform 2, UnitProtKB Q61548-2). AP180 consists of an N-terminal lipid binding ANTH domain and a C-terminal assembly domain with multiple clathrin binding motifs, as defined by Morgan *et al.* [29]. (B) The C-terminal domain can be further divided into the CLAP sub-domain and an unnamed ∼16 kDa C-terminal sub-domain. Nominal position of aa residues at domain/sub-domain boundaries and calculated isoelectric points [42] for each domain are shown. (C) GST/GFP-AP180 sequences used in pull-downs and transfections. Clathrin binding motifs are shown as solid blue circles.

AP180 has two known domains: the ANTH domain and a C-terminal assembly domain which binds both AP2 and clathrin and is responsible for the assembly activity of AP180 (Fig. 1A) [39]. However, it was previously noted in rat [40] and mouse AP180 [41] that the isoelectric point of different parts of AP180 varies widely and is suggestive of three, rather than two, domains. In support of this, our analysis of mouse AP180 isoform 2 shows that AP180 can be divided into three domains according to isoelectric point [42] (Fig. 1B). The ANTH domain of AP180 is moderately basic (pI 8.2). The central motif-rich CLAP domain (287–729) is very acidic (pI 3.5). Also, the CLAP domain consists of 62% of either Ala, Pro, Ser or Thr residues and contains an uncharged segment of 59 aa residues [41]. The ∼16 kDa C-terminal sub-domain is highly basic (pI 10.1), but has few charged residues overall.

We truncated AP180 to make sequences that contain one or more of these domains to examine clathrin binding (Fig. 1C). M1 and C1 are the isolated CLAP and ∼16 kDa C-terminal sub-domains, respectively. Two other truncated sequences divide AP180 and the CBMs in approximately one half (N½ and C½). The Lafer group first defined the AP180 CBMs as having a core D(L/I)(L/F) motif and established a relationship between the rate of *in* *vitro* clathrin assembly and the number of AP180 CBMs [29]. The number of CBMs differs in each of our AP180 sequences (Fig. 1C): C1 (730–896, 1 CBM), C½ (493–896, 7 CBMs), N½ (1–530, 5 CBMs), M1 (287–730, 11 CBMs) and full length (FL) (1–896, 12 CBMs). Note that the highly degenerate CBM in C1, with a core D_772_LQ_774_, is least likely to be a legitimate CBM. A sequence with only the ANTH+CLAP was also produced (1–730, 11 CBMs). These AP180 sequences were used to test the relationship between the location of CBMs and the level of *in* *vitro* clathrin binding.

**Figure 2:**
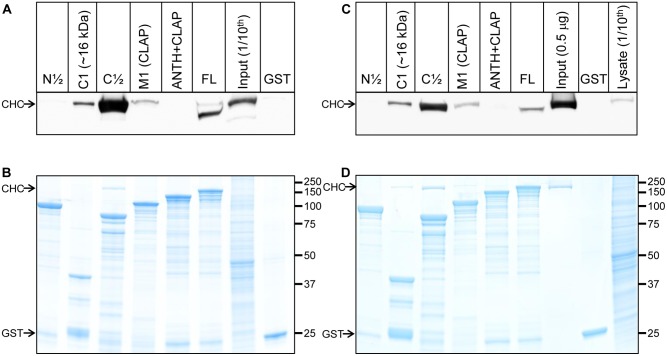
Binding of clathrin to different parts of AP180. GST-AP180 truncated sequences were used in pull-downs with synaptosome lysate (A–B) and purified clathrin (C–D). Each sample was resolved by SDS-PAGE gel, transferred onto nitrocellulose membrane and probed with a CHC antibody. (A) Western blot of pull-down using synaptosomes lysate, resolved on a large format gel. One representative Western blot from three independent experiments is shown. (B) One tenth of the amount of protein used to produce (A) was loaded onto a mini-gel and stained with Coomassie blue to show the relative amount of GST fusion protein used as bait. (C) Western blot of pull-down using purified clathrin, resolved on a mini-gel. One representative Western blot from two independent experiments is shown. For comparison, the same amount of lysate loaded as input in (A) was loaded into the right-most lane of (C). (D) Forty percent of the amount of protein used to produce (C) was loaded onto a mini-gel and stained with Coomassie blue. The migration of CHC and GST is indicated by arrows.

Glutathione S-transferase (GST) tagged FL AP180 and truncated sequences were used in pull-down experiments with rat brain synaptosome lysate (Fig. 2A and B) or purified clathrin from bovine brain (Fig. 2C and D) in 1% triton X-100. Binding of clathrin was assessed by Western blotting using an antibody against clathrin heavy chain (CHC). The amount bound to each AP180 sequence is shown in Fig. 2A and C. The relatively equal amount of GST fusion protein used as bait is shown in Fig. 2B and D. Compared to FL AP180, C1 and M1 bound clathrin relatively weakly (Fig. 2A and C), despite that M1 was predicted to bind clathrin well, since it has 11 CBMs. N½ has 5 CBMs but did not detectably bind clathrin. Notably, C1 bound clathrin without any proven or clearly recognised CBM to support clathrin interaction. Furthermore, C½ bound more strongly to clathrin than FL AP180. Note that the high load of bait and similar SDS-PAGE migration of GST-AP80 FL and CHC can distort and push the CHC band to a lower apparent molecular mass for that particular lane only (GST-AP180 FL pull-down with synaptosomes lysate resolved on a large format SDS-PAGE gel). Some, but not all, of the data agrees with previous work using clathrin purified from brain [29], [30], [39] (see Discussion). A similar relative amount of clathrin was pulled down from both the lysate and the solution with only purified bovine clathrin, indicating that the relative amount of clathrin pulled-down by each fragment was not a result of gain/loss of accessory proteins.

The clathrin from bovine brain was purified using a method that separates clathrin from other proteins [21], [39]. The purity of the clathrin was confirmed by SDS-PAGE analysis and mass spectrometry (Fig. S1). Therefore, it is unlikely that contaminating endocytic accessory proteins influenced the pull-down with purified clathrin.

The failure of N½, ANTH+CLAP and the CLAP domain to use their CBMs (5, 11 and 11 respectively) to bind clathrin well suggests that an important clathrin binding element is missing from these fragments or that there are inhibitory elements in the CLAP domain that prevent strong direct clathrin binding. Overall, there was a lack of correlation between bound clathrin and the number of available CBMs and sequences that retained the C-terminus were better clathrin binders.

### The ∼16 kDa C-terminal sub-domain is required for clathrin interaction with AP180

**Figure 3:**
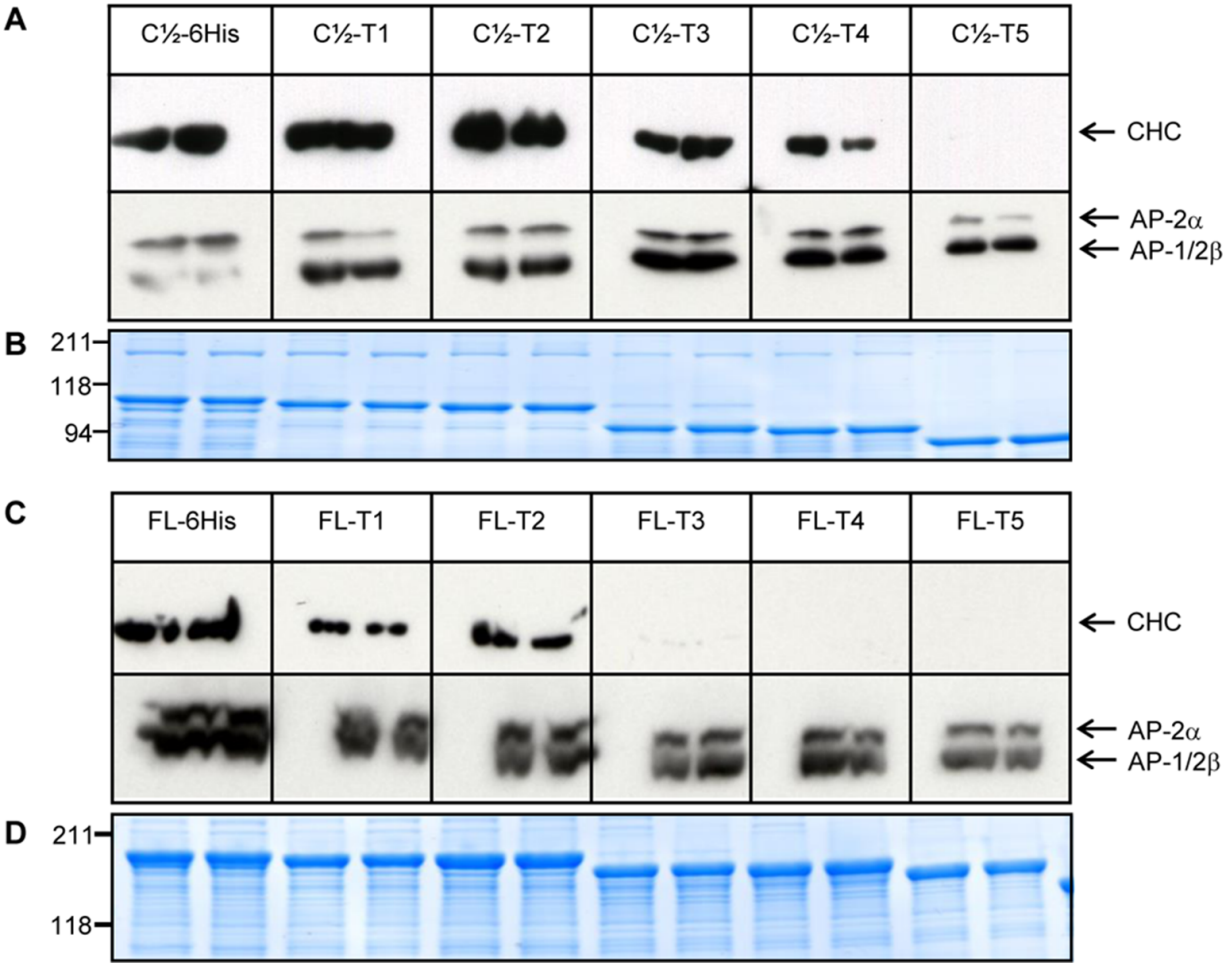
The ∼16 kDa C-terminal sub-domain is required for clathrin binding. (A) C½, C½-T1, C½-T2, C½-T3, C½-T4 and C½-T5 were used in pull-downs with rat brain lysate (separate rats for each duplicate lane). Each sample was run on a large format 10% SDS-PAGE gel, transferred onto nitrocellulose membrane and probed with a CHC antibody. (B) One eighth of the amount used to produce (A) was loaded onto a mini-gel and stained with Coomassie blue to show the relative amount of GST fusion protein used as bait. (C) AP180 FL, FL-T1, FL-T2, FL-T3, FL-T4 and FL-T5 were used in pull-down experiments with rat brain lysate and probed with a CHC antibody. (D) One eighth of the amount used to produce (C) was loaded onto a mini-gel and stained with Coomassie blue to show the relative amount of GST fusion protein used as bait.

To investigate clathrin binding to the ∼16 kDa C-terminal sub-domain of AP180, we progressively truncated the C-terminus of both FL AP180 and the C½ sequence, which bound strongest to clathrin. First, C½ was truncated to produce C½-T1 to -T5 (Fig. 1) and used in pull-down experiments with rat brain lysate. Western blot detection of CHC showed that clathrin binding was affected by truncation (Fig. 3A). The first truncation to show an obvious decrease in CHC binding was C½-T3, suggesting that the AP180 786–896 sequence has a role in clathrin binding, despite lacking known CBMs. Binding was similarly reduced when most of the ∼16 kDa C-terminal sub-domain was absent in C½-T4. Detectable clathrin binding was abolished in C½-T5, despite the presence of 5 CBMs. When FL AP180 was C-terminally truncated in the same way, clathrin binding was greatly reduced following the smallest truncation (FL-T1, Fig. 3C); however, we have also truncated a 6His tag which might have had some small influence on binding (see below). Clathrin binding was abolished using a relatively short truncation, FL-T3, despite this recombinant protein retaining all CBMs. The need for truncations of different length for FL and C½, to abolish clathrin binding, may reflect their different affinities for clathrin, prior to truncation (Fig. 2). We concluded that sequences in the ∼16 kDa C-terminal sub-domain are required for clathrin binding to the known CBMs.

One explanation for the abolished clathrin binding is that C-terminal truncation might induce the normally disordered/unfolded state of the AP180 CLAP and ∼16 kDa C-terminal sub-domains [30] into a folded state which prevents protein binding. To test this we re-probed our Western blot with anti-AP2α and anti-AP2β. Using anti-AP2α, we found that C½-T5 and FL-T3 were still able to bind AP2 with a small reduction in amount (compared to complete loss of CHC), demonstrating that these sequences were still available for protein-binding (Fig. 3A and C). Anti-AP2β gave similar results, but was less specific for the purpose of gauging AP2 binding, since this antibody also detects AP-1β. We conclude that the loss of clathrin binding was mainly due to the loss of the ∼16 kDa C-terminal sub-domain sequences.

**Figure 4:**
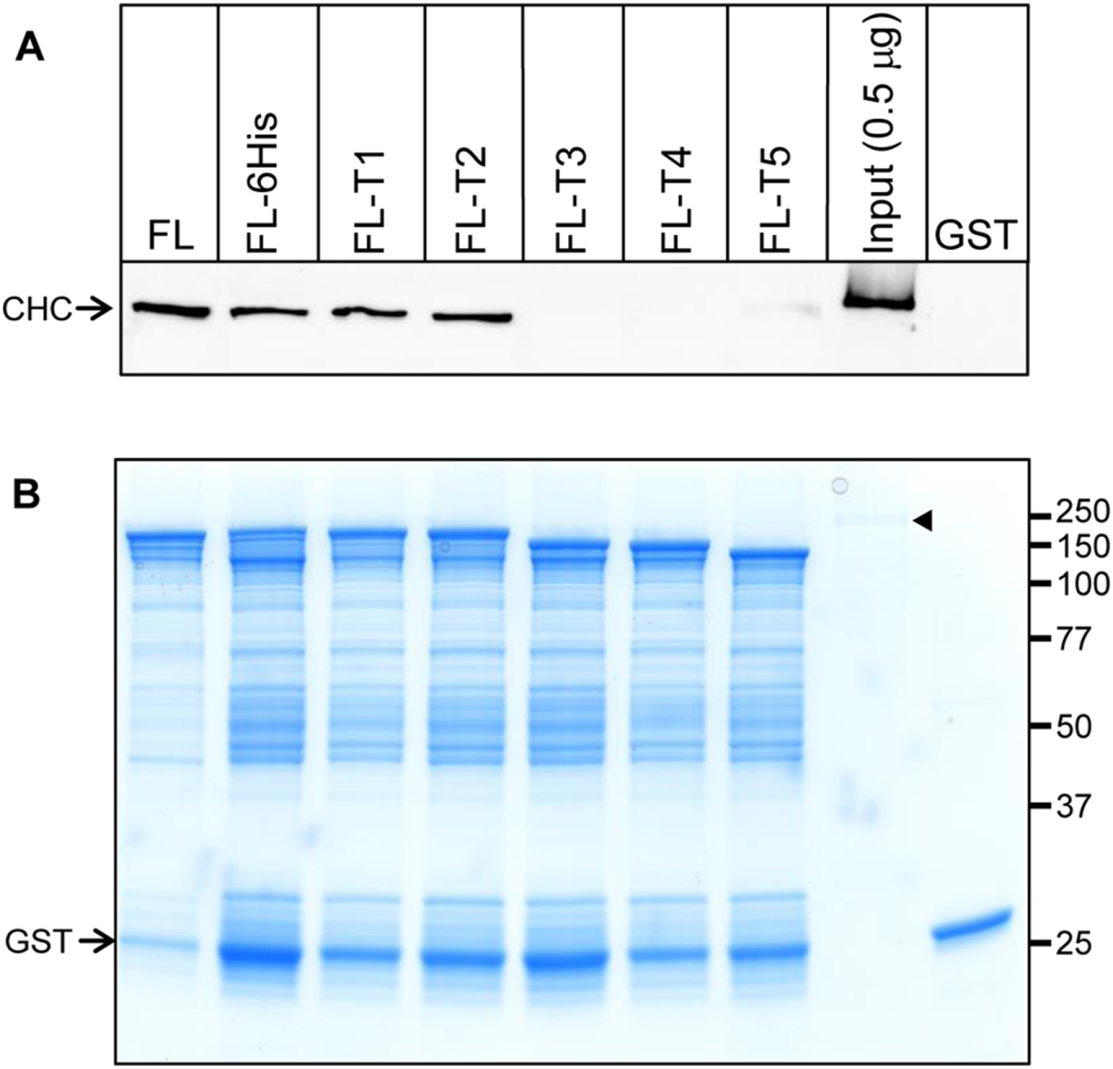
Purified clathrin binds to C-terminally truncated AP180 the same as clathrin from lysate. (A) GST tagged AP180 FL (codon optimised, see Methods), FL-6His, FL-T1, FL-T2, FL-T3, FL-T4 and FL-T5 were used in pull-downs with purified bovine clathrin as the only other protein present. The samples were resolved by SDS-PAGE mini-gel, transferred onto nitrocellulose membrane and probed with a CHC antibody. A representative Western blot from two independent experiments is shown. (B) Forty percent of the amount of protein used to produce (A) was loaded onto a mini-gel and stained with Coomassie blue to show the relative amount of GST fusion protein used as bait. The migration of CHC and GST is indicated by arrows. In (B), the migration of the weakly stained CHC (0.2 µg) is shown by an arrow head.

A possible explanation for the requirement of the ∼16 kDa C-terminal sub-domain was that a co-factor/accessory protein from the brain/synaptosome lysate promoted AP180-clathrin binding or interfered with binding to the truncated AP180. To rule this out we repeated the pull-down using only purified clathrin from bovine brain (Fig. 4). The Western blot for CHC (Fig. 4A) showed that FL AP180 had no difficulty binding clathrin in the absence of lysate. Furthermore, CHC binding became undetectable when using FL-T3 as bait, mirroring the result using lysate (Fig. 3C). We concluded that there was no evidence for a co-factor/accessory protein in the lysate that promotes binding of clathrin to the FL AP180 or interferes with binding to the truncated AP180.

Note that the FL sequence in Fig. 2 had a C-terminal 6His tag. Since C-terminal interaction with clathrin may be crucial, we removed the tag (Fig. 4A, lane 1). We also took the opportunity to codon optimise the plasmid sequence (see Methods) to potentially reduce the influence of AP180 fragments bound to the beads. After these two changes, the amount of clathrin bound to the de-tagged codon optimised FL was similar to the FL-6His (Fig. 4A). The codon optimised AP180 was used for all sequences in the pull-downs shown in Fig. 2. The result shown in Fig. 2 was the same when this experiment was done in triplicate with non-optimised 6His tagged FL and truncated AP180 (data not shown). No 6His tag or codon optimisation was used in mammalian cells. Thus, we have no reason to suspect that the 6His tag or protein expression fragments influenced our main conclusions. Codon optimisation was successful in reducing the level of GST-AP180 fragments in the bacterial expression (Fig. 4B *c.f.* lane 1 and 2).

### The effect of truncation of the ∼16 kDa C-terminal sub-domain on transferrin uptake

C-terminal AP180 sequences have been routinely used as a tool to inhibit CME [43] because the FL or C-terminal assembly domain are dominant negative when transfected into mammalian cells. These are “AP180-C” (530–915), which comprises the ∼16 kDa C-terminal sub-domain and approximately half the CBMs in the CLAP domain [28], and AP180 330–900, which is almost the entire C-terminal assembly domain of AP180 [44]. When over-expressed in cell cultures, both sequences nearly completely block CME, presumably by sequestering and mis-localising all of the cytosolic clathrin [44]. To test the hypothesis that the AP180 ∼16 kDa C-terminal sub-domain plays an important role in clathrin binding, GFP-tagged C½ and FL truncated sequences were transfected into COS-7 cells. We used a Tfn-Alexa 594 nm emission dye uptake assay to measure the effect of these AP180 sequences on CME.

**Figure 5:**
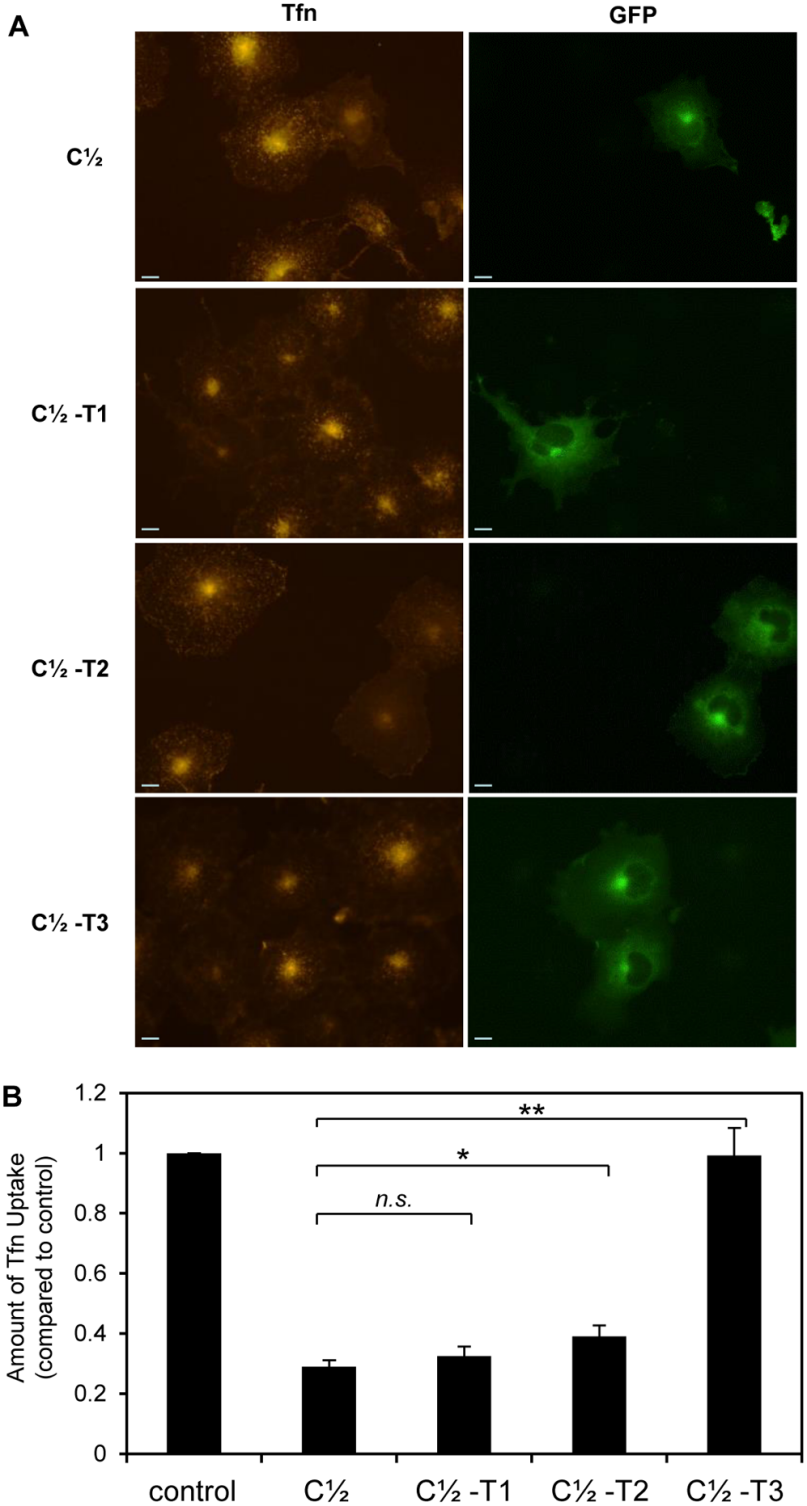
The effect of C-terminal truncation of C½ on transferrin uptake. (A) Uptake of transferrin (conjugated to Alexa Fluor 594 nm, red) in COS-7 cells transfected with GFP-C½, -C½-T1, -C½-T2 and -C½-T3 (green). Scale bars represent 20 µm. (B) Amount of transferrin uptake was quantified using Metamorph for *n* = 30 cells. Data is expressed as the average fraction of the transferrin uptake in untransfected (control) cells ± S.E.M (note: control intensity was normalised to 1). A t-test was done comparing each C-terminally truncated sequence to C½ (*n.s.*, not significant; *, p<0.05; **, p<0.01).

Transfection of GFP-C½ had a dominant negative effect on CME (Fig. 5), as observed previously with the similar AP180-C sequence [28]. GFP-C½-T1 and GFP-C½-T2 transfected cells exhibited a slight recovery in Tfn uptake when compared to GFP-C½, but only GFP-C½-T2 was significantly recovered. However, GFP-C½-T3 did not inhibit Tfn uptake. These observations correlate with clathrin binding (Fig. 3A) and suggest that GFP-C½-T3 has reduced ability to sequester CHC and inhibit CME.

**Figure 6:**
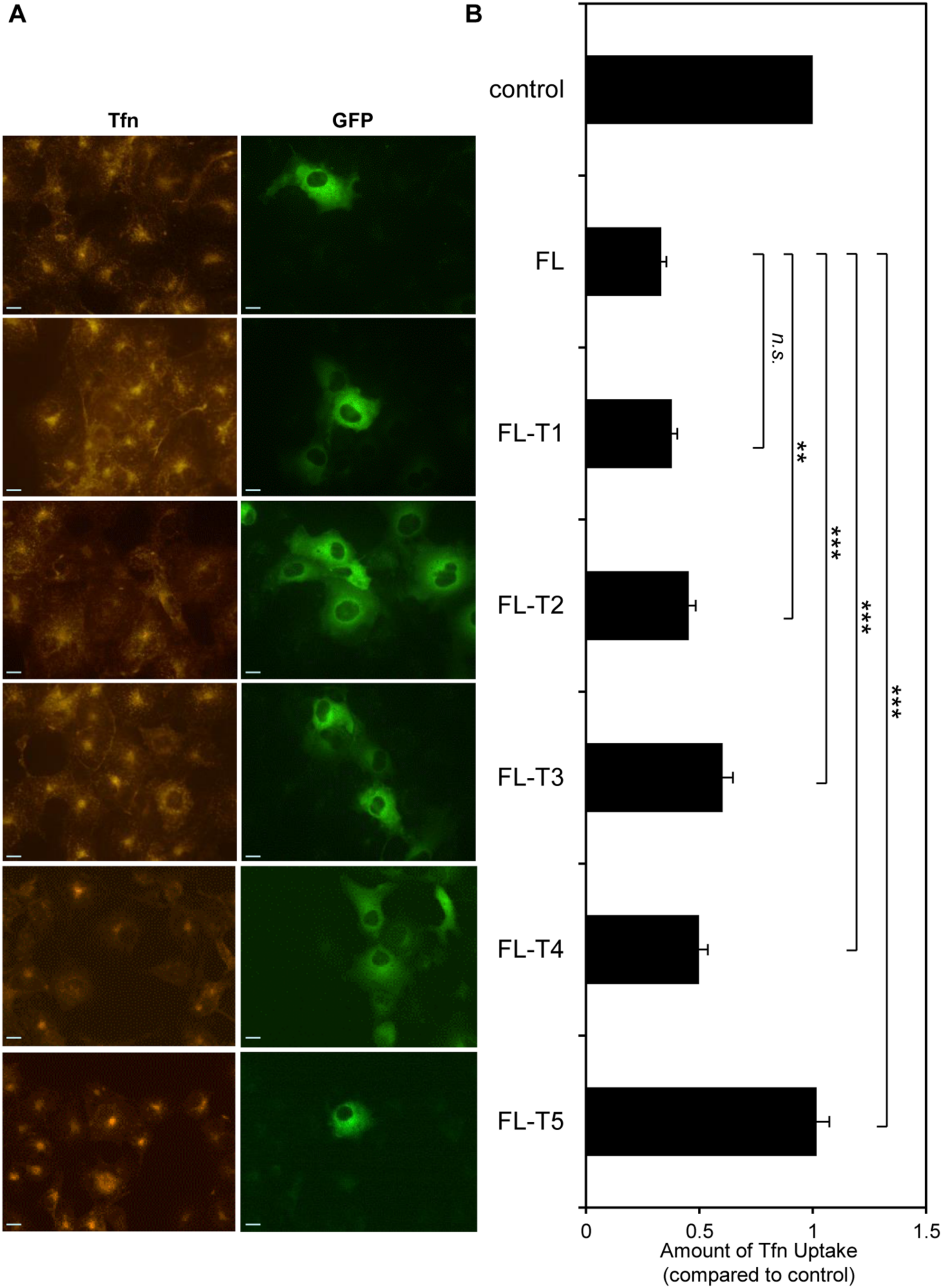
The effect of C-terminal truncation of FL on transferrin uptake. (A) Uptake of transferrin (conjugated to Alexa Fluor 594 nm, red) in COS-7 cells transfected with GFP-AP180 FL, -FL-T1, -FL-T2, -FL-T3, -FL-T4 and -FL-T5 (green). Scale bars represent 20 µm. (B) Amount of transferrin uptake was quantified using Metamorph for *n* = 30 cells. Data is expressed as the average fraction of transferrin uptake in untransfected (control) cells ± S.E.M (note: control intensity was normalised to 1). A t-test was done comparing each truncated sequence to FL (*n.s.*, not significant; **, p<0.01; ***, p<0.001).

FL AP180 had a dominant negative effect on CME (Fig. 6) similar to C½, as shown previously [28], [44]. The inhibitory effect was reduced when FL was truncated to FL-T3 (Fig. 6), but not completely abolished as observed above with C½-T3 (Fig. 5B). Inhibition of CME was no longer evident with transfection of GFP-FL-T5. This requirement of a larger truncation in FL, to abolish CME inhibition, suggests that the FL sequences had a larger residual dominant negative effect than the C½ sequences. The reduced binding of clathrin to FL-T1 and FL-T2, relative to FL-6His, as seen in pull-downs (Fig. 3) correlated with the observable *ex* *vivo* effects which were significant for FL-T2 (Fig. 6). Therefore, the extreme C-terminus of AP180 may contain a site that influences clathrin binding, but there are other unidentified sites in the ∼16 kDa C-terminal sub-domain that are crucial. Overall, the data suggests that multi-valent binding via the ∼16 kDa C-terminal sub-domain is critical for AP180-clathrin interaction in cells.

**Figure 7:**
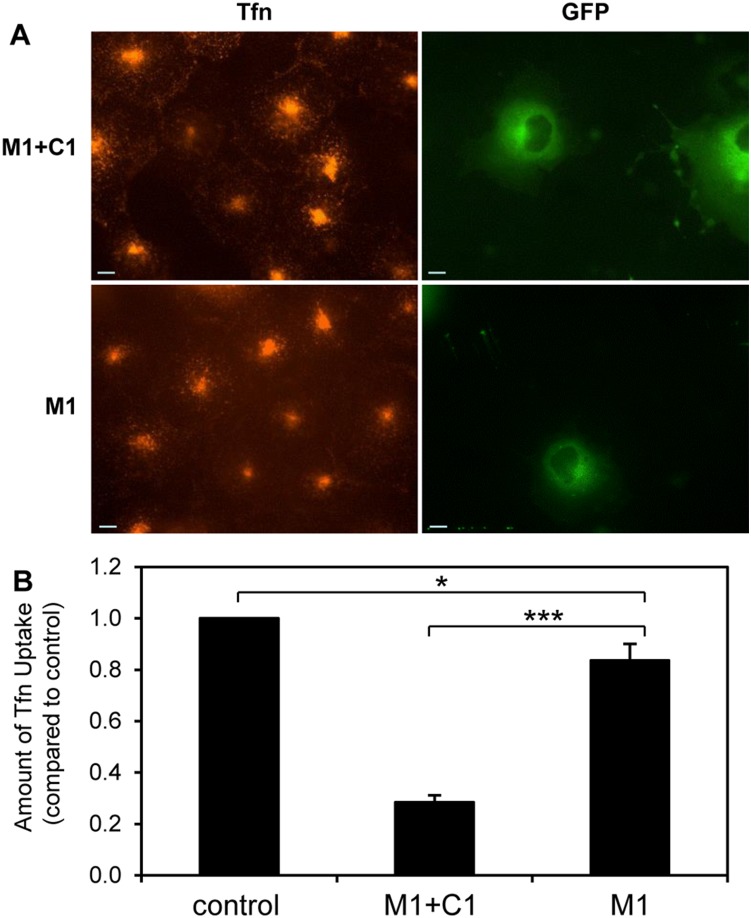
The effect of ∼16 kDa C-terminal sub-domain deletion from the AP180 C-terminal assembly domain. (A) Uptake of transferrin (conjugated to Alexa Fluor 594 nm, red) in COS-7 cells transfected with GFP-M1+C1 and GFP-M1 (green). Scale bars represent 20 µm. (B) Amount of transferrin uptake quantified using Metamorph for *n* = 30 cells. Data is expressed as the average fraction of transferrin uptake in untransfected (control) cells ± S.E.M (note: control intensity was normalised to 1). A t-test was done comparing M1 to M1+C1 (***, p<0.001) and M1 to control (*, p<0.05).

We examined Tfn uptake using sequences correlate with the three domains predicted by isoelectric points (*i.e.*, the ANTH domain and the CLAP and ∼16 kDa C-terminal sub-domains). Transfection of GFP-M1+C1 (CLAP + ∼16 kDa C-terminal sub-domain) was compared to GFP-M1 (CLAP) (Fig. 1 and 7). GFP-M1+C1 had a dominant negative effect, similar to FL, C½ and previous work [44] (Fig. 7A & B). GFP-M1 only mildly inhibited Tfn uptake (∼15%) (Fig. 7A & B), despite its possession of 11 CBMs. This confirms that the absence of the ∼16 kDa C-terminal sub-domain inhibits clathrin-AP180 interaction *ex* *vivo*.

### Interaction of the ∼16 kDa C-terminal sub-domain with clathrin *ex* *vivo*

**Figure 8:**
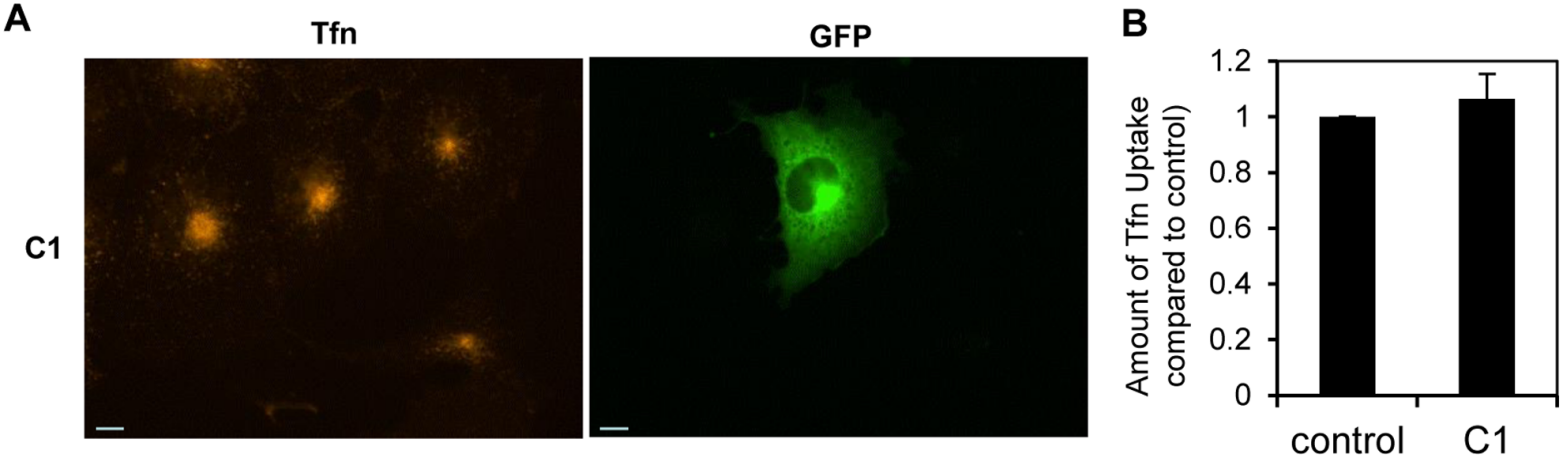
The effect of C1 on transferrin uptake. (A) Uptake of transferrin (conjugated to Alexa Fluor 594 nm, red) in COS-7 cells transfected with GFP-C1 (green). (B) Amount of transferrin uptake was quantified using Metamorph for *n* = 30 cells. Data is expressed as the average fraction of transferrin uptake in untransfected (control) cells ± S.E.M (note: control intensity was normalised to 1). There was no significant difference between transfected and untransfected cells (t-test).

Despite that the ∼16 kDa C-terminal sub-domain is crucial for clathrin binding to AP180 *in* *vitro* and *ex* *vivo*, the isolated ∼16 kDa C-terminal sub-domain bound clathrin relatively weakly *in* *vitro* (GST-C1, Fig. 2A and C). We examined whether a similar result would be expected for the isolated ∼16 kDa C-terminal sub-domain in the Tfn uptake assay. GFP-C1 did not cause a dominant negative effect (GFP-C1, Fig. 8A and B), like FL, M1+C1 or C½. We conclude that C1-clathrin binding is weak and unable to sequester clathrin without partnership with the CLAP domain.

**Figure 9:**
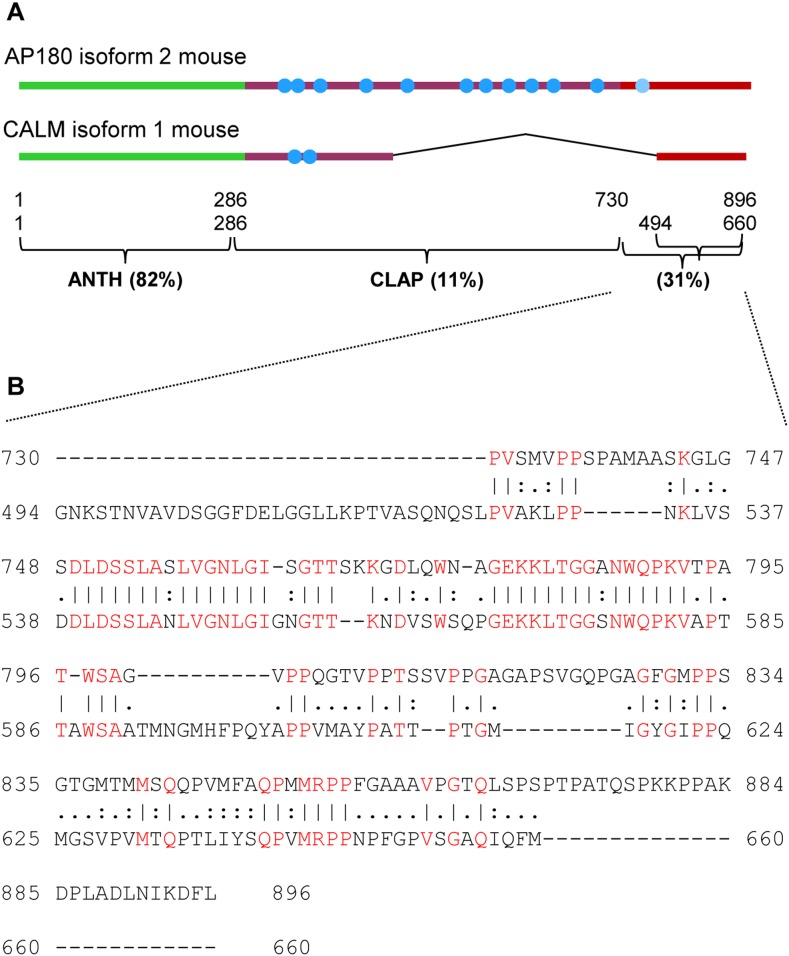
Alignment and similarity of the AP180 and CALM ∼16 kDa C-terminal sub-domains. (A, B) Similarity between domains of mouse AP180 isoform 2 and mouse CALM isoform 1 using EMBOSS Needle pair wise sequence alignment [56]. Parameters for sequence alignment were default (EBLOSUM62). Clathrin binding motifs, as defined by Morgan *et al.* [29], within AP180 and CALM are shown as blue circles in (A). The percentage of identical residues for each domain/sub-domain is shown. (B) Identical (“|”) and similar (“:”) aa residues are indicated for the ∼16 kDa C-terminal sub-domain. Identical aa residues are also in red (identity 30.7%, similarity 40.6% and gaps 42.5%).

*In* *vitro* binding and Tfn uptake studies on C-terminally truncated CALM indicates that clathrin binding near the C-terminus of CALM is crucial and involves multiple sites [35], [36]. Sequence alignment of AP180 and CALM [45] (Fig. 9) indicates that the ∼16 kDa C-terminal sub-domain also exists in CALM with a 31% sequence identity, which was higher than the identity between the CALM and AP180 CLAP domain (11%).

## Discussion

### Clathrin binding is highly dependent on the ∼16 kDa C-terminal sub-domain

We have defined three domains of AP180 based on isoelectric point [40], [41] and investigated the affinity of AP180 fragments for clathrin in pull-downs, using a similar approach to previous work [29], [30], [39]. The previous studies also used truncated AP180, but were done exclusively with clathrin purified from bovine brain, whereas our study is the first to use triton X-100 soluble lysates. Our results were mostly in agreement. The isolated CLAP domain (M1) did not bind clathrin as efficiently as FL (Fig. 2A and C). This was observed previously, without explanation, using a similar sized central sequence [39]. We found that the isolated ∼16 kDa C-terminal sub-domain could weakly bind clathrin. A similar ∼16 kDa C-terminal sequence was previously reported to bind clathrin cages, but not clathrin triskelia [39]. Our CLAP fragment did not bind as well as a similar fragment reported previously [39]. The slight differences in binding are likely due to different buffer conditions. These findings add to previous work that showed the CLAP domain and a ∼16 kDa C-terminal sequence could not assemble clathrin and bound purified clathrin much more weakly than the assembly domain they were derived from [39]. Since there was no correlation between the number of CBMs in each fragment and clathrin binding, we concluded that a previously unexplained factor is crucial for clathrin binding.

The loss of clathrin binding after N- or C-terminal truncation is unlikely to be explained by the loss of an accessory protein, since the pull-downs showed the same result for clathrin binding from lysate (Fig. 2A and 3) and from a solution of purified clathrin (Fig. 2C and 4).

A circular dichroism study of AP180 found that the assembly domain has little or no secondary structure [34]. The CLAP sub-domain must also be disordered, which should make each CBM equally available for binding. Therefore, it is counterintuitive that clathrin was relatively inefficiently pulled-down by the CLAP domain (M1, Fig. 2A and C) and the overexpressed CLAP domain could not sequester clathrin *ex* *vivo* (Fig. 7B), like FL, M1+C1 and C½. The CLAP is available to bind proteins other than clathrin, as demonstrated by the negligible effect of C-terminal truncation on AP2 binding (Fig. 3). Since clathrin bound better to sequences that retained the C-terminus (FL, C½, and M1+C1), we concluded that the CLAP is unable to bind clathrin as well as FL because it lacks the crucial ∼16 kDa C-terminal subdomain, *i.e.* the C-terminal sub-domain is the missing factor that explains weak binding of clathrin to the isolated CLAP domain.

It is also counter to expectations that C½ bound clathrin stronger than FL AP180. Our C½ sequence has not previously been studied. Before now, no fragment of AP180 has been observed to bind clathrin stronger than the FL sequence. Perhaps the different CBMs have different affinities for clathrin and the CBMs closer to the C-terminal part of the CLAP domain have a stronger affinity. However, clathrin binding to CBMs is multi-valent [29], [30]. Thus, clathrin should be able to access multiple low/high affinity CBMs in the CLAP domain equally as well as it should for the FL sequence, if there were no other factors influencing binding. Another possibility is that AP180 has sequences in the N-terminal half of the CLAP domain that inhibit clathrin binding. The weakness of a model involving auto-inhibition is that is requires there to be a part of AP180 that prevents access to CBMs, which implies that structural elements exist in the CLAP domain. Since there is no evidence for secondary structure, an auto-inhibition model remains unlikely. Alternatively, C½ may have an advantage over FL in how its disordered sequence folds onto the structured clathrin domains. Work is in progress to define how the sub-domains of AP180 interact with clathrin.

The isolated CLAP domain bound slightly better than the ANTH+CLAP suggesting that the ANTH inhibits binding to the CLAP, perhaps by directly binding the CLAP domain. This would align AP180 with a recent discovery for AP2, where a clathrin binding subdomain in the β2 hinge was found to be auto-inhibited by binding to the membrane interacting AP2 core [18]. However, ANTH-CLAP binding is unlikely to be a significant effect because the isolated CLAP is still a very weak clathrin binder in the absence of the ANTH. In contrast, the isolated clathrin binding sub-domain of µ2 binds clathrin well.

### The ∼16 kDa C-terminal sub-domain of AP180 is essential for CME

Progressive truncation of the ∼16 kDa C-terminal sub-domain from C½ or FL AP180 abolished or greatly reduced clathrin binding *in* *vitro* and *ex* *vivo*. The FL sequence required more truncation than C½ and both sequences also required different length truncations *ex* *vivo* than they did *in* *vitro*, before clathrin binding was reduced. This was most evident in the Tfn uptake following expression of FL-T4, which did not follow the trend of gradual relief from the dominant negative effect. This might be explained by interactions of these sequences with AP2, chaperones or other co-factors and their differing availability in the lysate vs the compartmentalised COS-7 cellular environment. For example, AP180 binds both clathrin and AP2 *in* *vitro* [17], however, transfected AP180 does not sequester or mis-localise AP2 the same way it affects clathrin when overexpressed in cells [44]. Another possibility is that one experiment correlates with binding while the other correlates with binding and some assembly activity. The pull-downs were not perfect predictors of the level of cellular clathrin sequestration. However, all experiments support the conclusion that truncation of the ∼16 kDa sub-domain reduces clathrin binding, *in* *vitro* and *ex* *vivo*.

The ∼16 kDa C-terminal sub-domain has weak clathrin binding *in* *vitro* and non-existent binding *ex* *vivo* (Fig. 2 and 8). Although it cannot strongly interact with clathrin alone, our truncation data supports the existence of at least two sites in the ∼16 kDa C-terminal sub-domain that mediate clathrin binding. The AP180 ∼16 kDa C-terminal sub-domain has aa sequences that are conserved within CALM (Fig. 9). The conserved aa residues in CALM and AP180 may harbour clathrin binding sites. The human CALM 530–583 sequence was found to be important for CALM sequestration of clathrin [36]. Also, Tebar et al. [35] established that at least two *in* *vitro* clathrin binding sites exist within 414–652 of human CALM. Thus, AP180 and CALM are likely to have a similar dependence on multiple sites within the ∼16 kDa C-terminal sub-domain that directly or indirectly mediate binding to clathrin.

The gene for CALM (*PICALM*) was identified as a risk gene in late onset Alzheimer’s disease [46] and is known to participate in gene fusions that cause leukemia [47], [48]. The gene for AP180, *SNAP91*, has been linked to mood-incongruent psychotic bipolar disorder [49]. Our observations of AP180-clathrin binding may add to hypotheses of how impaired AP180 and CALM function impacts on disease [12]. Since the leukemia gene fusions result in the partial loss and replacement of the ∼16 kDa C-terminal sub-domain [50], our results suggest there could be a loss of clathrin binding function, dependent on the breakpoint, in addition to the known detrimental effects of protein fusion [37], [38].

In summary, the ∼16 kDa C-terminal sub-domain of AP180 is a separate functional sub-domain required for efficient CLAP domain-clathrin binding and is supported by homology with CALM.

## Materials and Methods

### Construction and expression of truncated AP180 sequences

The AP180 sequences depicted in Figure 1 were sub-cloned into an N-terminal GST vector and or an N-terminal GFP vector (EGFP-C1). The GST vector was modified to all expression of C-terminal 6His tag (made by inserting 5′-GGCCGCATGAAAACCTGTATTTTCAGGGCCATCATCATCATCATCATTAATAA-3′ and 3′-CGTACTTTTGGACATAAAAGTCCCGGTAGTAGTAGTAGTAGTAATTATTCCGG-5′ into the Not1 cleavage site of the pGEX-6P-1 vector), but 6His affinity purification was not used for this study. Wild type GST-AP180 (mouse isoform 2) sequence was provided by Prof. Eileen Lafer (University of Texas Health Science Center, San Antonio, Texas, USA). C1, C½, M1+C1, M1 and FL were made by PCR with the following sense primers: 5′-GTCGACATGTCGGGCCAAACGCTCACGG-3′ (FL), 5′-TCGAGCGGCCGCGCCAAGAAATCCTTGATGTTAAGATCCGCTAACGGGTCC-3′ (C1, C½, M1+C1, FL), 5′-GTCGACTCCGGGCAGCCTGCCCCTG-3′ (C1), 5′-GTCGACAGCAATGAAGCCACCTGAGAC-3′ (C½), 5′-GTCGACGGAAAGAAACCTGGAAACAATGAAGGATCTGG -3′ (M1+C1, M1) and 5′-TCGAGCGGCCGCGCGGATGGTGCCATGGTTGGCA-3′ (M1). C½-T1, C½-T2, C½-T3, C½-T4, C½-T5, FL-T1, FL-T2, FL-T3, FL-T4, FL-T5 were made by PCR with the following sense primers: 5′-GCGGATCTTAACTAAAAGGATTTC-3′ (C½-T1, FL-T1), 5′-GAAATCCTTTTAGTTAAGATCCGC-3′ (C½-T1, FL-T1), 5′-CCAGCCAAGTAACCGTTAGCGG-3′ (C½-T2, FL-T2), 5′-CCGCTAACGGTTACTTGGCTGG-3′ (C½-T2, FL-T2), 5′-GCTGGGGAGTAAAAGCTGACTGG-3′ (C½-T3, FL-T3), 5′-CCAGTCAGCTTTTACTCCCCAGC-3′ (C½-T3, FL-T3), 5′-CCTCGGAAGTTAACTTGACTCG-3′ (C½-T4, FL-T4), 5′-CGAGTCAAGTTAACTTCCGAGG-3′ (C½-T4, FL-T4), 5′-CCAGCTCAGTAAAACCTGCTGC-3′ (C½-T5, FL-T5), 5′-GCAGCAGGTTTTACTGAGCTGG-3′ (C½-T5, FL-T5). FL AP180 was codon optimised for optimal expression in *Escherichia coli* (Data S1) and cloned into pGEX-6P-1. Deletion mutations to produce ANTH+CLAP, N½, C1, M1 and C½ were done by GenScript (Piscataway, New Jersey, USA). GST constructs were transformed into *Escherichia coli* strains BL21 or JM109 by heat shock. The recombinant proteins were expressed for four hours after induction and bound to glutathione (GSH)-Sepharose beads.

### Antibodies

Anti-clathrin (X22) was from Abcam (Cambridge, UK). Anti-AP2α and anti-adaptin β were from BD transduction laboratories (Palo Alto, CA, USA). All horse-radish peroxidase (HRP)-conjugated secondary antibodies were from DAKO (Carpinteria, CA, USA.

### Pull-down experiments

Purified clathrin was a kind gift of Prof. Eileen Lafer (University of Texas Health Science Center, TX, USA) and was purified as described previously [21], [39]. In this method, fractions from gel filtration of enriched clathrin [21] were examined by SDS-PAGE for contaminating protein bands and further by Western blot for AP180 and AP-2 contamination [39]. Only those clathrin containing fractions with little or no detectable contaminants were pooled. We also confirmed the absence of endocytic accessory proteins in the purified clathrin by mass spectrometry (See mass spectrometry method below and Fig. S1).

Purified clathrin was centrifuged at 75,000 rpm for 10 min prior to use and only the supernatant used in pull-downs. Brains were extracted from rats following decapitation. All animal care and use complied with local legislation and procedures were performed in accordance with institutional guidelines and approved by the CMRI/CHW Animal Care and Ethics Committee. The brains were washed briefly with ice-cold PBS and homogenised in 20 mM Tris (pH 7.4), 150 mM NaCl, 1% Triton X-100, 1 mM EDTA, 1 mM EGTA, 20 µg/ml leupeptin, 1 mM phenylmethylsulfonyl fluoride (PMSF), and 1 tablet of complete EDTA-free protease inhibitor per 10 ml (Roche, Castle Hill, NSW, Australia) and 1∶100 dilution of phosphatase inhibitor cocktail set II (Merck Millipore (Calbiochem), Kilsyth, Vic, Australia). The homogenate was centrifuged at 22,973×*g* for 20 min at 4°C. Synaptosomes were prepared from rat brains as described previously [51]. The supernatant was incubated at 4°C for 1 h with GSH-Sepharose beads (GE Healthcare Life Sciences, Rydalmere, NSW, Australia) coated with various GST-AP180 full length and truncated proteins. Each pull-down involved the use of ∼10 µg of bead-bound protein to extract protein from ∼100 mg of rat brain lysate, ∼100 mg synaptosomes lysate or 2 µg of purified clathrin from bovine brain. The pull-down with purified clathrin was done in the same lysis buffer and incubated at 4°C for 4 h. The beads were washed extensively with a solution of 20 mM Tris (pH 7.4), 1 mM EDTA, 1 mM EGTA and 1 tablet of complete EDTA-free protease inhibitor per 10 ml, then eluted in 2× concentrated SDS-PAGE sample buffer. A fraction of the pulled-down proteins were resolved by SDS-PAGE in 1 mm thick 10% or 12% large gels (20 cm) or mini-gels (Protean II or Mini-Protean, Bio-Rad, Gladesville, NSW, Australia), followed by either Coomassie Blue staining or Western blot analysis using antibodies described above. All duplicate/triplicate pull-down experiments were done using material from separate rat brains with equal amounts of GST fusion protein bait.

### Mass spectrometry

Three separate lots of 2 µg of purified clathrin were precipitated by the chloroform-methanol method [52], dissolved in 50 µl of 50 mM triethylammonium bicarbonate and digested with 0.1 µg trypsin (TrypZean, Sigma-Aldrich, Castle Hill, Australia) at 37°C for 16 hrs. One half (1 µg) of each digested clathrin solution was desalted using C18 material packed into a pipette tip, eluted, dried, redissolved in a 0.1% formic acid solution and analysed by LC-MS/MS. The LC-MS/MS was done as described previously [53] using a Dionex Ultimate 3000 nanoHPLC and Velos orbitrap mass spectrometer (Thermo Fisher Scientific, Scoresby, Vic, Australia) with some minor changes. Briefly, the 5 µl sample was loaded onto a 30 cm 75 µm column pack with ReproSil-Pur 120 C18-AQ 3 µm beads (Dr Maisch, Germany) at 400 nl/min for 20 min and then the peptides were separated at 250 nl/min using a gradient from 2% phase A (0.1% formic acid in water) to 35% phase B (0.1% formic acid, 9.9% water and 90% acetonitrile) in 30 min and then to 100% B in 5 min. During each cycle of MS detection, the top seven peptides above 5000 counts in a 30,000 resolution orbitrap MS scan were selected for fragmentation by an MS/MS scan at 7,500 resolution in the orbitrap.

The raw MS files were processed using MaxQuant 1.5.0 [54]. The bovine reference proteome from UniProtKB was used (UP000009136, Bos taurus, July 8 2014, 23842 entries). All MaxQuant values were default except that there was no fixed modification of cysteines, the minimum peptide length was 6, FTMS data was recalibrated, the minimum peptides was 2, the minimum “razor + unique peptides” was 2 and the minimum unique peptides was 1. The intensity based absolute quantification (iBAQ) [55] method was enabled to estimate the relative amount of each protein detected (not absolute, despite the name). This method essentially uses the sum of peptide intensities for a protein and divides this by the theoretical number of tryptic peptides so that proteins of different size are more comparable. The text output of the MaxQuant software, including all the parameters used, is supplied as Data S2. Proteins that did not have an iBAQ value for each of the three clathrin digestions were discarded. The iBAQ values for the remaining proteins were averaged and a standard error of the mean was determined using MS Excel.

### Cell culture

COS-7 cells (*Cercopithecus aethiops*, kidney, ATCC, Manassas, Virginia, USA) were grown in Dulbecco's modified Eagle's medium (DMEM) supplemented with 10% fetal bovine serum (FBS). GFP-AP180 plasmids were transfected into COS-7 cells using electroporation and the Invitrogen Neon Transfection System according to manufacturer’s instructions and plated immediately on cover slips coated with poly-D-lysine in DMEM with 10% FBS. Transfected cells were harvested or assayed 24 h post-transfection.

### Transferrin uptake endocytosis assay

The cells were serum-starved in DMEM with no FBS at 37°C for 3 h. Transferrin (Tfn)-A594 (5 µg/µl, Invitrogen, Eugene, Oregon, USA) was added and the cells were incubated at 37°C for 20 min. Plates were placed on ice to arrest Tfn uptake. After three washes with PBS, the cells were fixed immediately with 4% PFA for 15 min at room temperature followed by three washes with PBS. Cover slips were mounted on slides for examination using an epifluorescence microscope (Olympus IX81) and visualised using a Hammamatsu Orca-ERG CCD digital camera (Hammamatsu City, Japan). Images were captured in three channels: red (Tfn conjugated to Alexa Fluor 594 nm), green (GFP-AP180) and blue (DAPI, DNA) at the same time. Average Intensity (AI) of Tfn uptake per cell was quantified using the Metamorph software (version 7.7.0.0). The calculation for the amount of Tfn uptake in a transfected cell was done as such using the red channel: Amount of Tfn uptake = AI (transfected cell) – AI (background)/[AI (non-transfected cell) – AI (background)]. The AI of Tfn for 30 cells was measured for each transfected GFP-fusion. The mean, S.E.M and significance (t-test) was calculated (Microsoft Excel).

## Supporting Information

Figure S1 **Assessment of the purity of clathrin from bovine brain by SDS-PAGE and mass spectrometry.** A. SDS-PAGE analysis and Coomassie blue staining of clathrin purified from bovine brain. The amount of clathrin loaded in each lane is indicated (determined by spectrophotometry [22]). Contaminating SDS-PAGE protein bands were low in intensity compared to clathrin heavy and light chains. B. The purified clathrin was digested with trypsin and analysed by mass spectrometry (see Methods, n = 3, intensity is the average +/− SEM). The relative amount of each protein was determined using the label-free iBAQ method [55]. Of all the known synaptic vesicle endocytosis [4] or clathrin mediated endocytosis proteins [1], only trace amounts of actin, heat shock cognate 71 kDa and AP180 were detected.
(TIF)

Data S1 **Codon optimised sequence of FL-AP180 for expression in** ***Escherichia coli*** **.** Mouse AP180 codons were optimised to remove those used rarely in *Escherichia coli.* Also, we reduced, but did not minimise, the variety of codons used to produce the same aa. The smallest base pair change was made for the required for silent mutation. Codons of frequency 1 were replaced with codons of higher frequency that code for the same aa.
(TXT)

Data S2 **Contains the tabulated output of the MaxQuant software from processing the LC-MS/MS data of the tryptic digestion of purified clathrin.**
(ZIP)
