# Supplementary figures and images for: The ∼16 kDa C-Terminal Sequence of Clathrin Assembly Protein AP180 Is Essential for Efficient Clathrin Binding
Source: PLoS One. 2014 Oct 20;9(10):e110557. doi: 10.1371/journal.pone.0110557 (PMC4203807; doi:10.1371/journal.pone.0110557)

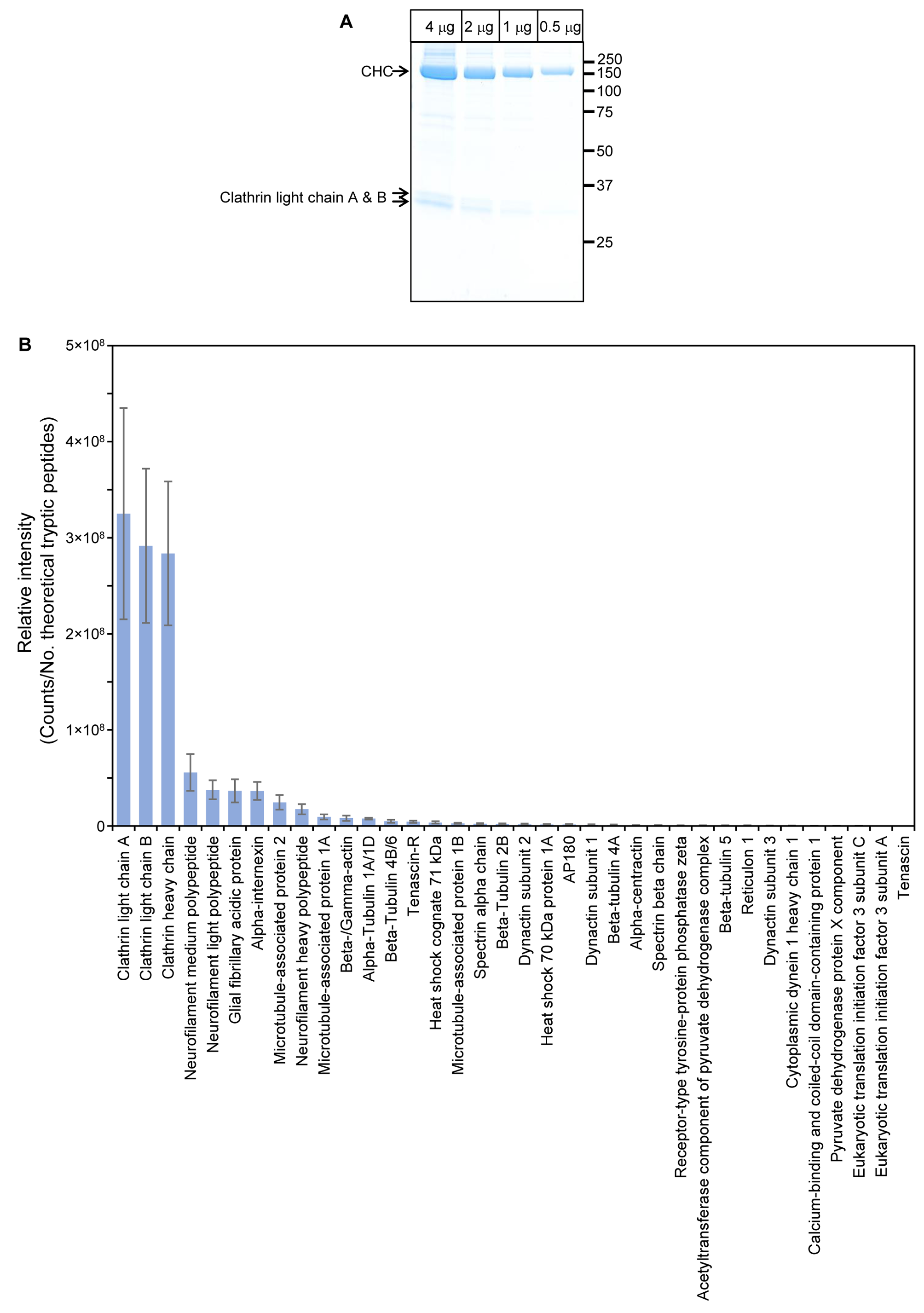

Supplement: Figure S1 — Assessment of the purity of clathrin from bovine brain by SDS-PAGE and mass spectrometry. A. SDS-PAGE analysis and Coomassie blue staining of clathrin purified from bovine brain. The amount of clathrin loaded in each lane is indicated (determined by spectrophotometry [22]). Contaminating SDS-PAGE protein bands were low in intensity compared to clathrin heavy and light chains. B. The purified clathrin was digested with trypsin and analysed by mass spectrometry (see Methods, n = 3, intensity is the average +/− SEM). The relative amount of each protein was determined using the label-free iBAQ method [55]. Of all the known synaptic vesicle endocytosis [4] or clathrin mediated endocytosis proteins [1], only trace amounts of actin, heat shock cognate 71 kDa and AP180 were detected. (TIF) [file pone.0110557.s001.tif]
